# Visible-Light Driven TiO_2_ Photocatalyst Coated with Graphene Quantum Dots of Tunable Nitrogen Doping

**DOI:** 10.3390/molecules24020344

**Published:** 2019-01-18

**Authors:** Xiong Sun, Hui-Jun Li, Nanquan Ou, Bowen Lyu, Bojie Gui, Shiwei Tian, Dongjin Qian, Xianying Wang, Junhe Yang

**Affiliations:** 1 School of Materials Science and Technology, University of Shanghai for Science and Technology, No. 516 Jungong Rd., Shanghai 200093, China; sunxiong1993@163.com (X.S.) huijunli0701@126.com (H.-J.L.); ounanquan@163.com (N.O.); bowenlyu0324@163.com (B.L.); bojie_gui@163.com (B.G.); tianshiwei17@163.com (S.T.); jhyang@usst.edu.cn (J.Y.); 2 Department of Chemistry, Shanghai Key Laboratory of Molecular Catalysis and Innovative Materials, Fudan University, No. 220 Handan Rd., Shanghai 200433, China; djqian@fudan.edu.cn

**Keywords:** N-doping GQDs, titanium dioxide, surface modification, photocatalytic, RhB degradation

## Abstract

Nitrogen doped graphene quantum dots (NGQDs) were successfully prepared via a hydrothermal method using citric acid and urea as the carbon and nitrogen precursors, respectively. Due to different post-treatment processes, the obtained NGQDs with different surface modifications exhibited blue light emission, while their visible-light absorption was obviously different. To further understand the roles of nitrogen dopants and N-containing surface groups of NGQDs in the photocatalytic performance, their corresponding composites with TiO_2_ were utilized to degrade RhB solutions under visible-light irradiation. A series of characterization and photocatalytic performance tests were carried out, which demonstrated that NGQDs play a significant role in enhancing visible-light driven photocatalytic activity and the carrier separation process. The enhanced photocatalytic activity of the NGQDs/TiO_2_ composites can possibly be attributed to an enhanced visible light absorption ability, and an improved separation and transfer rate of photogenerated carriers.

## 1. Introduction

Photocatalytic reactions driven by solar light are a promising method for solving the existing environmental and energy problems. Development of new photocatalysts, and optimizing their performance to realize photodegradation of organic pollutants under visible light irradiation, has attracted worldwide attention. So far, various semiconductors, including metal oxides, sulfide and oxygen nitrides, and graphite carbon nitrides, have been successfully synthesized, among which TiO_2_ has been reported as an efficient and suitable photocatalyst of great practical potential due to its stability, high specific surface area, and the ability to produce charge carriers under UV irradiation [1,2,3,4]. However, its wide bandgap energy (3.2 eV) hinders the effective utilization of visible light, resulting in low photocatalytic efficiency under solar light irradiation, which would limit further practical application [5,6]. On the other hand, photo-generated charge carriers with a high recombination rate could also lead to low photocatalytic efficiency. To narrow the band gap and enhance the charge carrier’s separation rate, coupling P25 TiO_2_ with other semiconductor quantum dots (QDs) (such as CdS [7] and CdSe [8]) has been attempted, due to their size-adjustable bandgap, high extinction coefficience and multiple exciton generation. Meanwhile, in most reported semiconductor quantum dots/TiO_2_ composites, visible light can be effectively utilized and the recombination of carriers can be inhibited [9,10]. However, most of these semiconductor quantum dots are highly unstable or toxic under light irradiation, which inevitably impairs their usefulness and causes environmental problems.

Recently, graphene quantum dots (GQDs), as new stars in the family of carbon nanomaterials, have attracted intensive attention in the field of photocatalysis [11,12,13,14]. Compared with conventional semiconductor quantum dots, zero-dimensional GQDs, composed of several layers of sp^2^ hybridized cellular carbon structures, is environmentally friendly. Besides, GQDs synthesized via hydrothermal methods usually contain rich functional groups, such as carboxyl and hydroxyl groups, which are beneficial for GQDs to form cohesive bonds with TiO_2_ [15]. The bonding could then promote fast electron transfer in the interfacial region of the GQDs/TiO_2_ composite structure, thus inhibiting carrier recombination [16,17,18,19,20]. Despite these advantages, the absorption band of GQDs is mainly confined in the UV region, which limits their solar energy utilizing efficiency in photocatalysis. Heteroatom doping is an effective method to solve this problem by changing the electron density and adjusting their optical and electrical properties [21,22,23,24]. Among these dopants, nitrogen doping not only preserves the intrinsic properties of GQDs, such as a large surface area, quantum size effect, and good biocompatibility, but also develops unique optical-electric properties [25]. Through adjusting the N doping amounts, different percentages of the C–N configuration in the structure can further influence the electron delocalization and charge carrier density. These particular performances have endowed NGQDs with more possibilities in constructing new visible-light driven photocatalysts.

Herein, GQDs and NGQDs have been successfully prepared via a hydrothermal method using citric acid and urea as the carbon and nitrogen precursors, respectively. Due to different post-treatment processes, the obtained NGQDs with different surface modification exhibited blue light emission while their visible-light absorption has an obvious difference. To furtherly understand the roles of nitrogen dopants and N-containing surface groups, the photocatalytic degradation activities of different composites under visible light irradiation were measured and are discussed.

## 2. Results and Discussion

### 2.1. TEM and HRTEM Studies

**Figure 1:**
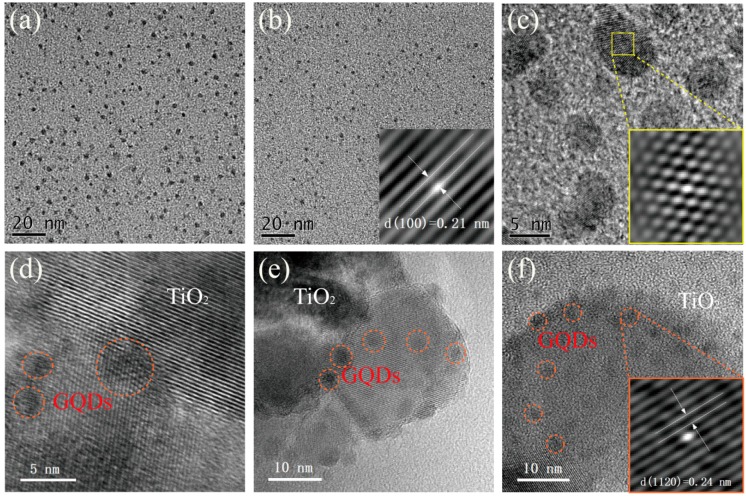
TEM images of (**a**) GQDs, (**b**) y-NGQDs and (**c**) g-NGQDs. The inset in (**b**) is the HRTEM image of y-NGQDs. The inset in (**c**) is the autocorrelated HRTEM lattice images recorded from the corresponding selected areas. The HRTEM images of (**d**) GQDs/TiO_2_, (**e**) y-NGQDs/TiO_2_ and (**f**) g-NGQDs/TiO_2_ composites. The insert in (**f**) is the autocorrelated HRTEM lattice image of the pristine g-NGQDs/TiO_2_ composites recorded from the corresponding selected areas.

Figure 1 shows the TEM (Transmission electron microscope) and HRTEM (High resolution transmission electron microscope) images of graphene quantum dots and their corresponding composites. Figure 1a–c shows the TEM images of GQDs, y-NGQDs and g-NGQDs, respectively. All samples had a relatively uniform particle distribution with an average lateral size from 3 nm to 8 nm. The inset in Figure 1b shows the autocorrelated HRTEM lattice image of pristine y-NGQDs recorded from the corresponding selected areas (marked by a yellow rectangle). Clear lattice fringes of 0.21 nm were assigned to the (100) plane of NGQDs, revealing that the crystallinity of NGQDs is similar to graphene. The autocorrelated HRTEM lattice image (insert in Figure 1c) shows a clear hexagonal honeycomb structure of g-NGQDs [26]. The TEM images of the composites (Figure 1d–f) provide direct evidence for successful anchoring of GQDs and NGQDs on the surface of TiO_2_ nanoparticles (marked by the red circles). The autocorrelated HRTEM lattice image (inset in Figure 1f) showed clear lattice fringes of 0.24 nm, which could be assigned to the (1120) plane of NGQDs.

### 2.2. XRD, FT-IR and XPS Studies

**Figure 2:**
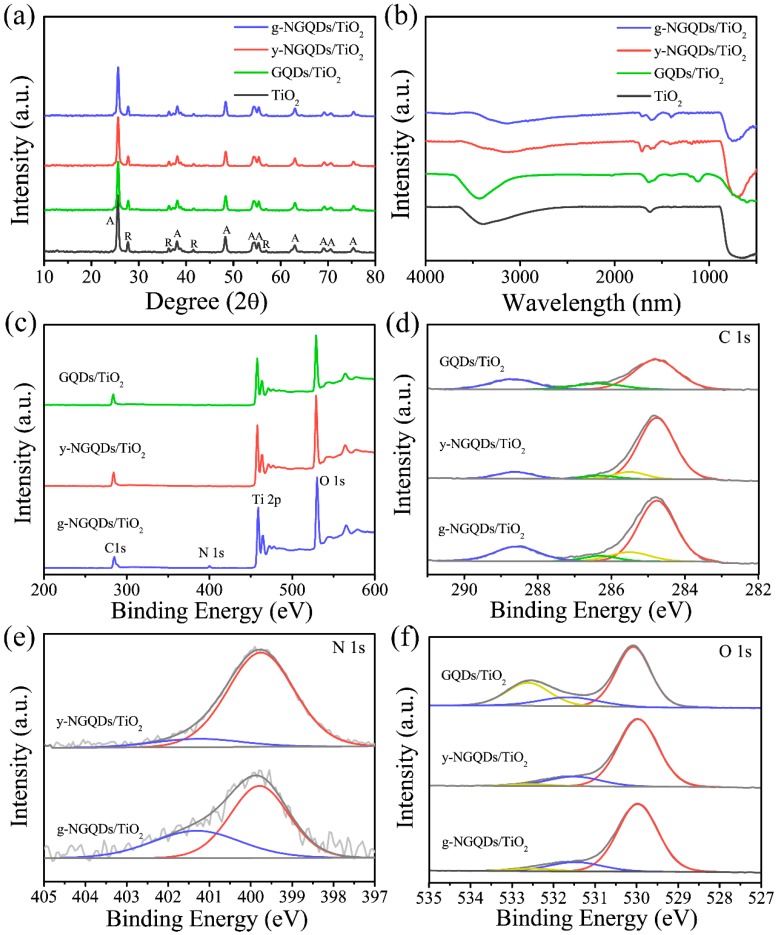
(**a**) XRD patterns (A represents the anatase phase, R represents the rutile phase) and (**b**) FT-IR (Fourier transform infrared)spectra of TiO_2_, GQDs/TiO_2_, g-NGQDs/TiO_2_ and y-NGQDs/TiO_2_ composites; (**c**) XPS (X-ray photoelectron spectroscopy) survey spectra, (**d**) C 1s, (**e**) N 1s and (**f**) O 1s high-resolution spectra of GQDs/TiO_2_, g-NGQDs/TiO_2_ and y-NGQDs/TiO_2_ composites.

The structure of the composites was further characterized using XRD (X-ray powder diffraction), FT-IR (Fourier transform infrared), and XPS (X-ray photoelectron spectroscopy) spectra. As shown in Figure 2a, all samples exhibited the same diffraction peaks assigned to anatase TiO_2_ and rutile TiO_2_ [27]. No characteristic diffraction signals of NGQDs were detected in the composites, suggesting that the NGQDs did not affect the crystalline structure of TiO_2_ due to the small contents of NGQDs in the composites [28]. The FT-IR spectra of the pristine TiO_2_, GQDs/TiO_2_, g-NGQDs/TiO_2_, and y-NGQDs/TiO_2_ are shown in Figure 2b. Obviously, additional vibration absorption peaks of NGQDs in the composites could be observed. Besides absorption peaks including C=C vibrations at ~1570 cm^−1^, C–H vibrations at ~1198 cm^−1^ and C–OH vibrations at ~1398 cm^−1^ shown in all composites, there are C–N vibrations at 1480 cm^−1^ and C=N vibrations at 1706 cm^−1^ for g-NGQDs/TiO_2_ and y-NGQDs/TiO_2_ composites, which furtherly confirms the successful combination of NGQDs with TiO_2_.

To study the difference of chemical states between g-NGQDs/TiO_2_ and y-NGQDs/TiO_2_, XPS spectra were measured, as shown in Figure 2c–e. Besides the signals of Ti 2p, C 1s, and O 1s, weak N 1s signals were detected in the g-NGQDs/TiO_2_ and y-NGQDs/TiO_2_ composites which is indicative of the nitrogen dopants in NGQDs (Figure 2c). Figure 2d shows the C 1s high-resolution spectra of g-NGQDs/TiO_2_ and y-NGQDs/TiO_2_ compositions. The C spectra were deconvoluted into four peaks, including a C=C peak with a binding energy of ~284.6 eV, a C–N peak with a binding energy of ~285.4 eV, a C–OH peak with a binding energy of ~286.0 eV, and an O−C=O peak with a binding energy of ~288.5 eV [29]. The bonding composition reflected that NGQDs were modified with oxygen-rich functional groups on the graphene edge sites. Meanwhile, compared to y-NGQDs/TiO_2_ composites, the g-NGQDs/TiO_2_ composites exhibited higher proportion of C–N, C–OH and O−C=O surface groups (shown in Table 1). The N 1s high-resolution spectra of g-NGQDs/TiO_2_ and y-NGQDs/TiO_2_ are revealed in Figure 2e. The two peaks centered at 399.8 eV and 401.3 eV are assigned to NH_2_ and N–C bonding, respectively. However, the percentage of N–C bonding was significantly reduced in y-NGQDs/TiO_2_ composites. This could be due to the post-treatment process where the nitrogen-containing organic species were thermally decomposed. The O 1s high-resolution spectra of the two composites (Figure 2f) are fitted with three Gaussian peaks centered at 530.0 eV, 531.4 eV and 532.5 eV. The peaks at ~530.0 eV and 532.5 eV are attributed to the crystal lattice oxygen (Ti–O–Ti), and surface oxygen in the hydroxyl species and/or adsorbed water species, respectively. The binding energy of 531.4 eV could be assigned to the Ti–O–C bonding, demonstrating that NGQDs and TiO_2_ were probably combined through the Ti–O–C bonds [30]. It is worth noting that the Ti–O–C bonds proportion of g-NGQDs/TiO_2_ was higher than that of y-NGQDs/TiO_2_.

### 2.3. UV–Visible Absorption and PL Studies

**Figure 3:**
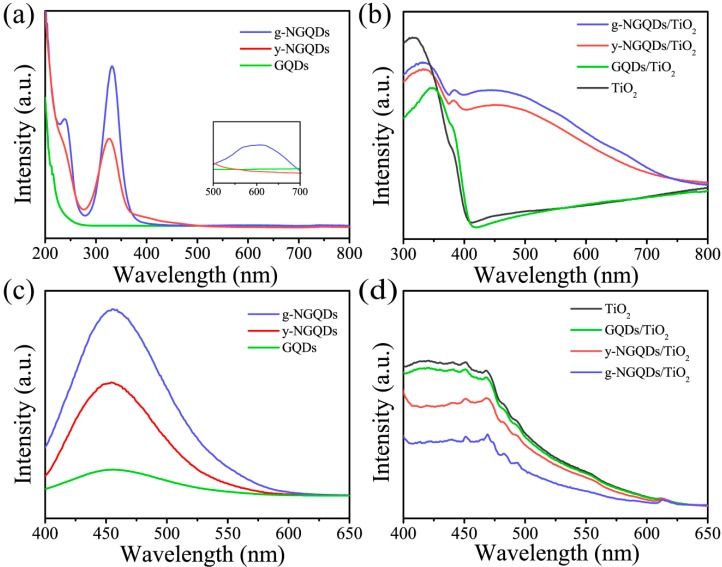
(**a**) UV-vis absorption spectra of the GQDs, g-NGQDs and y-NGQDs (The inset was an enlarged illustration of the original UV-vis absorption spectra ranged from 500 nm to 700 nm). (**b**) UV-DRS spectra of TiO_2_, GQDs/TiO_2_, g-NGQDs/TiO_2_ and y-NGQDs/TiO_2_ composites; (**c**) Comparison of PL spectra with excitation at 360 nm for GQDs, g-NGQDs and y-NGQDs; (**d**) Comparison of PL spectra with excitation at 360 nm for TiO_2_, GQDs/TiO_2_, g-NGQDs/TiO_2_ and y-NGQDs/TiO_2_ composites.

To explore the influence of structures and compositions on the optical and electrical properties of these composites, UV-vis absorption and PL (Photoluminescence) spectroscopy were carried out. Figure 3a shows the UV-vis absorption spectra of the GQDs, g-NGQDs and y-NGQDs. For g-NGQDs, two obvious excitonic absorption bands were observed, centered at about 238 and 332 nm, which could be attributed to the π-π* transition of aromatic C=C domains and the n-π* transition of C=O or C=N, respectively [31,32]. Notably, there is a blue shift of the absorption ascribed to n-π* transition from 332 nm in g-NGQDs to 327 nm in y-NGQDs. Furthermore, a distinct absorption peak around 500–700 nm appeared as the concentration of g-NGQDs solutions increased, while no absorption was observed for y-NGQDs and GQDs (the inset in Figure 3a). One suspects that the absorption was caused by the rich surface organic groups functioning as chromophores, which should have similar properties to the methyl blue dye [33,34,35,36]. UV-DRS was then employed to study the optical properties of different TiO_2_ composites. Pure TiO_2_ exhibited no absorption in the region above 400 nm, attributed to the intrinsic light absorption property of TiO_2_. Whereas, both g-NGQDs/TiO_2_ and y-NGQDs/TiO_2_ composites displayed an obvious increase in absorption in the visible light region compared to that of GQDs/TiO_2_ composites. This phenomenon indicates the importance of nitrogen doping in GQDs on broadening the photo-response range of TiO_2_ (Figure 3b). Also, the g-NGQDs/TiO_2_ showed stronger absorption than the y-NGQDs/TiO_2_. Based on the analysis of XPS spectra, it further confirmed the roles of nitrogen dopants and surface nitrogen-containing groups in light absorption enhancement [37]. These graphene structures might serve as an ideal hot carrier chromophore, allowing multiple electronic excitations under light irradiation and ultrafast equilibration of hot electrons (holes) to yield quasi-thermal distributions.

To understand the radiative recombination behavior of photoinduced charge carriers, PL emission spectroscopy is used to measure the defect states. The three GQD samples all show blue emission at 440 nm under excitation of 360 nm, mainly originated from the π→π* and n→π* transitions. The intensity of the emission peak of g-NGQDs is much higher than that of GQDs and y-NGQDs because more delocalized electrons exist in N-rich g-NGQDs. For primary P25 TiO_2_, the broad PL spectrum is a result of transition of electrons from different defect states. After anchoring with GQDs or NGQDs, the PL intensity of TiO_2_ decreases accordingly and the g-NGQDs/TiO_2_ composite shows the lowest intensity. The partial quenching of PL intensity is indicative of a lower carrier recombination rate and electron transfer from TiO_2_ to GQDs. Generally, GQDs can act as a light absorber and also effective conductive mediums for electrons to transfer to the surface of the composites [38]. Furthermore, the surface Ti–O–C bonding is beneficial for efficient interfacial charge transfer, which would play a vital role in the following dye degradation process [39].

### 2.4. Visible Light Photocatalysis Studies

**Figure 4:**
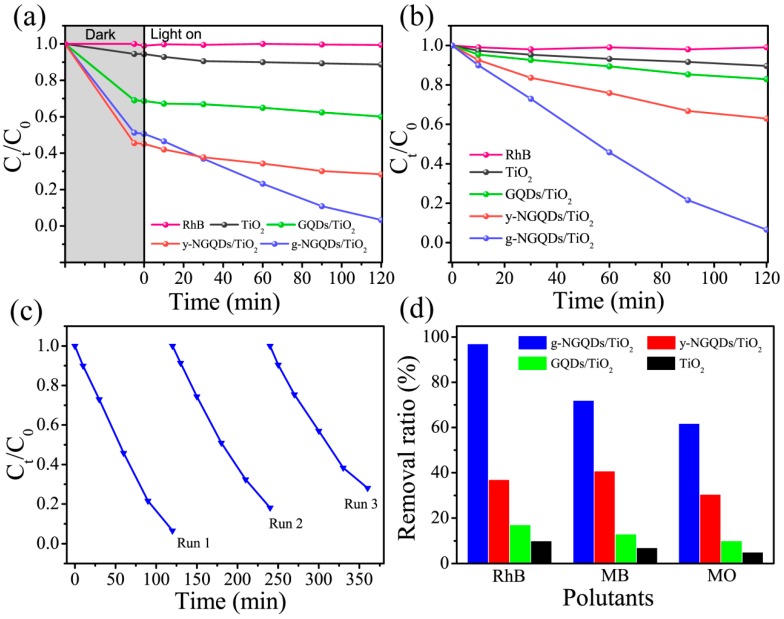
(**a**) RhB (Rhodamine B-Hyaluronate) absorption property and photodegradation activities under visible light irradiation (λ > 420 nm) of TiO_2_, GQDs/TiO_2_, g-NGQD/TiO_2_ and y-NGQD/TiO_2_ composites; (**b**) Visible-light driven photocatalytic degradation rates of RhB by TiO_2_, GQDs/TiO_2_, g-NGQD/TiO_2_ and y-NGQD/TiO_2_ composites; (**c**) Recycling test of the g-NGQDs/TiO_2_ for photocatalytic degradation of RhB aqueous solution under visible light irradiation (λ > 420 nm); (**d**) The removal ratios of RhB, MB and MO under visible light irradiation (λ > 420 nm) using TiO_2_, GQDs/TiO_2_, g-NGQD/TiO_2_ and y-NGQD/TiO_2_ composites.

The photodegradation activity of the NGQDs/TiO_2_ composites under visible light irradiation (λ > 420 nm) was evaluated, as shown in Figure 4. Figure 4a shows the trend of RhB (Rhodamine B-Hyaluronate) absorption in various samples under dark conditions. Compared with pure TiO_2_, a relatively high absorption ability was observed for the composites, which could be attributed to the GQDs of unique size effect and plenty of surface sites [40]. Blank reaction without catalysts implied that the self-degradation of RhB was negligible. When pure TiO_2_ photocatalyst was added into the dye solution, only 10% of RhB was degraded after 2 h of irradiation, owing to the wide bandgap nature of TiO_2_. Since P25 TiO_2_ exhibited no absorption in the region above 400 nm, the degradation could be attributed to the dye sensitization. Meanwhile, the GQDs/TiO_2_ composites showed a relatively low visible-light driven photocatalytic efficiency, of which about 17% of RhB was degraded after 2 h of irradiation. For g-NGQDs/TiO_2_ and y-NGQDs/TiO_2_ composites, 37% and 94% of RhB, respectively, could be degraded under the same reaction conditions. By comparing the degradation efficiency of pure TiO_2_ and NGQDs/TiO_2_, the contribution of the self-photosensitizer effect of RhB towards the photoactivity could be excluded. Based on the analysis of optical properties, the enhanced photocatalytic performance under visible light irradiation could be attributed to the strong absorption in the visible region (λ > 420 nm) and a relatively low carrier charge recombination rate.

Furthermore, the photocatalytic stability test of the g-NGQDs/TiO_2_ composites was measured, shown in Figure 4c. The photoactivity of the g-NGQDs/TiO_2_ decreased to 72% after 3 recycle times, which proved that the g-NGQDs/TiO_2_ sample is quite stable up to three repeated cycles. In addition, we tested the photocatalytic activity of the g-NGQDs/TiO_2_ composite for degrading methylene blue (MB) and methyl orange (MO) pollutants. Their corresponding photoactivities are provided in Figure 4d. It could be found that the photoactivity enhancement of the g-NGQDs/TiO_2_ composite has generality. The result demonstrated that GQDs play a significant role in enhancing visible-light driven photocatalytic activity, and nitrogen doping indeed influences both light absorption and the carrier separation process.

### 2.5. Photoelectrochemical Tests

**Figure 5:**
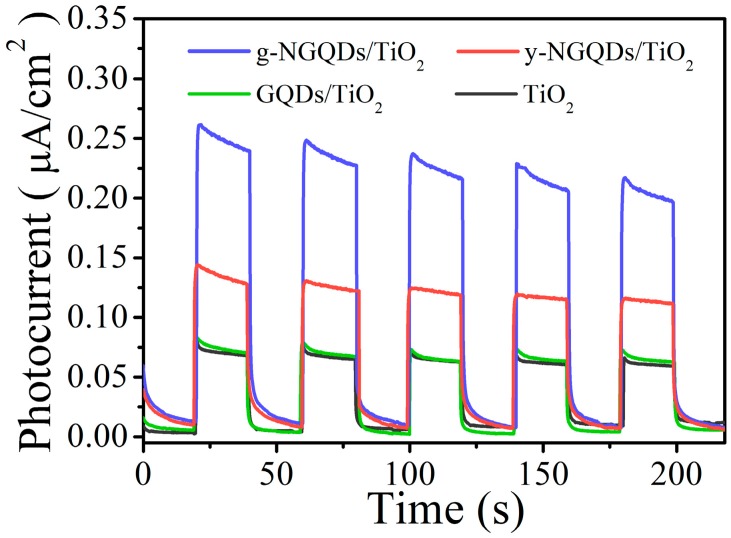
Transient photocurrent response spectra of TiO_2,_ GQDs/TiO_2_, g-NGQDs/TiO_2_ and y-NGQDs/TiO_2_ composites.

To deeply study the photogenerated charge separation and electron transfer performance of all samples, photocurrent responses were measured, shown in Figure 5. Each sample was recorded for several on–off cycles under visible-light illumination at a bias voltage of −0.2 V. For the TiO_2_ sample, a weak photocurrent signal was observed, probably due to the existence of oxygen vacancies induced by visible light irradiation [41]. All the composites showed higher photocurrent than bare TiO_2_, and the g-NGQDs/TiO_2_ composite exhibited the highest photocurrent, at about 2.5 times that of y-GQDs/TiO_2_ and 5.5 times that of GQDs/TiO_2_. The distinct enhancement of photocurrent under visible-light irradiation is consistent with the PL spectra, demonstrating the vital role of NGQDs in improving carrier separation and transfer rate, since N-GQDs possess good electrical conductivity [42].

### 2.6. Degradation Mechanism of RhB

**Scheme 1:**
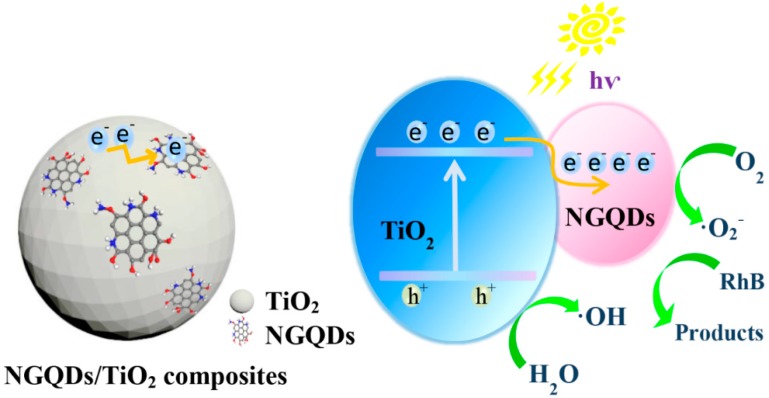
The mechanism for the enhanced photocatalytic activity of the NGQDs/TiO_2_ composites.

The possible mechanism of the enhanced photocatalytic activity of the NGQDs/TiO_2_ composites is illustrated in Scheme 1. NGQDs have discrete electronic levels and can function as a light absorber, allowing for hot electron generation, and their extended π-electron systems can facilitate the donor–acceptor contact through direct contact with the TiO_2_ surface. The coupling can also be enhanced by covalent Ti–O–C bonding between GQD and TiO_2_. Under irradiation of visible light, the coupling of NGQDs with TiO_2_ first enhanced the visible-light absorption compared to bare TiO_2_. Meanwhile, the electrons were excited from the VB (Valence Band) to CB (Conduction band) of P25 TiO_2_, and then transferred to the surface of NGQDs, whereas the holes in the VB reacted with sacrificial regents. Therefore, there is effective charge separation and the separated electrons would act as active species on the surface of NGQDs. The NGQDs in the composites have multiple functions and nitrogen doping plays a vital role in enhancing the overall photocatalytic performance.

## 3. Experimental Details

### 3.1. Chemicals and Reagents

All the chemicals are commercial available. All the chemicals were used without further purification. Citric acid monohydrate and potassium hydroxide were purchased from Sinopharm Chemical Reagent Ltd. (Shanghai, China). Urea was purchased from Shanghai Aladdin Biochemical Technology Co. (Shanghai, China), Ltd. P25 TiO_2_ was purchased from Degussa (Frankfurt, Germany).

### 3.2. Synthesis

#### 3.2.1. Synthesis of GQDs and NGQDs

Citric acid (1.68 g (8 mmol)) and 1.44 g (24 mmol) of urea were dissolved in 40 mL of water, and stirred to form a clear solution. Then the solution was transferred into a 50 mL Teflon lined stainless autoclave. The sealed autoclave was heated to 180 °C in 20 min and kept for 8 h. The obtained yellow solution of NGQDs was oxidized into a stable green solution after stirring for hours in air, which has been reported in our previous work [36]. The final product was collected by adding ethanol into the solution and centrifuging at 10000 rpm for 5 min. The green precipitates were dried in vacuum at 80 °C for 3 h. The green solids were easily re-dispersed into water, defined as g-NGQDs. When the green solid were further dried in vacuum at 180 °C for 3 h, brown solids were obtained, which could also be easily re-dispersed into water and show as a yellow solution, defined as y-NGQDs. GQDs without N doping were synthesized for comparison. Typically, 1.68 g (8 mmol) of citric acid and 1.34 g (24 mmol) of potassium hydroxide were dissolved in 40 mL of water, and stirred to form a clear solution. The sealed autoclave was heated to 180 °C in 20 min and kept for 8 h. The final product was collected by adding ethanol into the solution and centrifuging at 10000 rpm for 5 min. The precipitates were dried in vacuum at 80 °C for 3 h, and could be easily re-dispersed into water.

#### 3.2.2. Fabrication of GQDs/TiO_2_ and NGQDs/TiO_2_ Composites

**Scheme 2:**
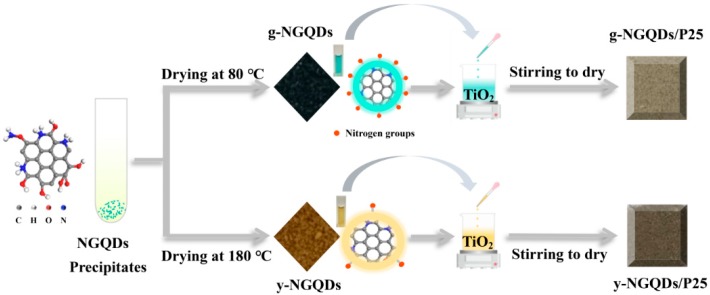
The synthesis procedures of g-NGQDs/TiO_2_ and y-NGQDs/TiO_2_ composites.

NGQDs were dissolved into 15 mL of water, and P25 was ultrasonically dispersed into 20 mL of ethanol. Then, the obtained NGQDs solution was dropped into the P25 TiO_2_ suspension under electromagnetic stirring at 60 °C. After drying out, the solids were washed with water and ethanol several times, and dried for 24 h at room temperature to obtain the NGQDs/TiO_2_ composites. The g-NGQDs/TiO_2_ show as light brown and y-NGQDs/TiO_2_ as dark brown. The synthesis procedures of g-NGQDs/TiO_2_ and y-NGQDs/TiO_2_ composites are shown in Scheme 2. The composites of GQDs without nitrogen doping show as white color.

### 3.3. Characterization

The X-ray powder diffraction (XRD) patterns were recorded using a Rigaku D/Max 2550 X-ray diffractometer with Cu-Ka radiation (γ = 0.15418 nm) in the range of 10° to 70° (2*θ*) at room temperature. The UV−vis absorption spectroscopy and UV–vis diffuse reflectance spectroscopy (DRS) were taken at room temperature on a UV-3150 spectrophotometer (Shimadzu, Kyoto, Japan). The photoluminescence (PL) spectroscopy was performed using a RF-5301pc fluorescence spectrometer (Shimadzu, Kyoto, Japan). The Fourier transform infrared (FT-IR) spectra were recorded on a Perkin Elmer GX spectrophotometer scanning from 4000 cm^−1^ to 400 cm^−1^ with a resolution of 4 cm^−1^. X-ray photoelectron spectroscopy (XPS) measurements were carried out on an ESCALAB 250 Xi (Thermo Scientific, Waltham, MA, USA) using non-monochromatized Mg–Kα X-ray as the excitation source. The binding energies for the samples were calibrated by setting the measured binding energy of C 1s to 284.60 eV.

### 3.4. Photocatalytic Test

Rhodamine B-Hyaluronate (RhB) was used as a hazardous organic pollutant. The evaluation of photocatalytic activity of the samples was performed on a home-built multizone photocatalytic reaction system, composed of eight parallel windows and a 500 W mid-pressure Hg lamp (Hg Arc lamp source; Shanghai Bilon Instrument Co., Ltd., Shanghai, P. R. China) equipped with a water filter and a 420 nm cut-off filter as a visible-light irradiation source. In a particular run, 10 mg of catalysts was added to 50 mL of a 20.0 mg L^−1^ RhB solution, 10.0 mg L^−1^ MB solution and 5.0 mg L^−1^ MO solution, respectively. Before irradiation, the suspension was magnetically stirred in the dark to reach the adsorption–desorption equilibrium of RhB, MB and MO on the surface of the photocatalyst. Approximately 3 mL of the suspension was collected at certain time intervals and then centrifuged at 11000 rpm for 5 min. The concentration change of the RhB, MB and MO solution was determined using a UV-vis spectrophotometer by monitoring its characteristic absorption peak at λ _max_ = 559 nm.

### 3.5. Recycling Test

The recycling test of the synthesized sample was done as follows. Typically, when a photocatalytic cycle was finished, the catalyst was separated from the reaction solution using high speed centrifuge and then directly employed in the next photocatalytic cycle after drying overnight in the vacuum at 80 ℃.

### 3.6. Photocurrent Measurements.

Photocurrent measurements were performed on an electrochemical analyzer (CHI 660C working station, CHI Shanghai, Inc., Shanghai, China), which has a conventional three-electrode configuration with a Pt foil as the counter electrode and an Ag/AgCl (saturated KCl) reference electrode, and 0.1 M Na_2_SO_4_ as the electrolyte. The working electrodes were fabricated by the modified electrophoretic deposition (EPD) method. Typically, 50 mg of the photocatalyst was mixed with 100 mL of isopropyl alcohol by sonication for 6 h, followed by adding 0.001 g of Mg(NO_3_)_2_·6H_2_O (≥98%; Alfa Aesar, Haverhill, MA, USA) into the suspension under ultrasonic for 1 h. A clean FTO (as cathode) facing the stainless steel anode was immersed into this suspension. The distance between the two electrodes was fixed at about 5 cm. The Mg^2+^-absorbed photocatalyst suspension was loaded in a quartz vessel as the electrolyte, and the electrophoresis process was performed at 60 V for 2 min. After the EPD process, the prepared electrodes were washed by ethanol and deionized water several times and dried at room temperature. A 350 W xenon lamp with a cut-off filter (λ > 420 nm) was used as light source and placed 20 cm away from the working electrode. The photocurrent of the samples was measured at −0.2 V.

## 4. Conclusions

In summary, GQDs and NGQDs of different surface modification were synthesized. Their corresponding composites with TiO_2_ were utilized to degrade RhB solutions under visible-light irradiation. The g-NGQDs/TiO_2_ composite showed the best degradation performance, about 9.4 times that of the pure P25 TiO_2_. The possible mechanism for the enhanced photocatalytic activity of the NGQDs/TiO_2_ composites is proposed, and can be attributed to enhanced visible light absorption ability and improved separation and transfer rate of photogenerated carriers. The NGQDs could function as a light absorber and electron transport medium. In addition, the Ti–O–C bonding is beneficial for efficient interfacial electron transfer from the CB band of P25 TiO_2_ to NGQDs. This work provides a new idea for the skilled application of GQDs with different surface modifications in constructing new efficient photocatalysts.

## Figures and Tables

**Table 1:** Percentage breakdown of different bonds within the XPS (X-ray photoelectron spectroscopy) high-resolution spectra calculated from Figure 2.

Bonds	Binding	Percentage Breakdown (%)		
	Energy (eV)	g-NGQDs/TiO_2_	y-NGQDs/TiO_2_	GQDs/TiO_2_
Ti-O-Ti	530.0	82.00	82.12	57.67
Ti-O-C	531.4	3.25	2.25	14.60
H_2_O	532.5	14.75	15.62	27.73
C-C	284.6	65.05	79.13	63.48
C–N	285.4	10.85	8.19	--
C-O	286.0	5.39	3.90	11.67
O-C=O	288.5	18.71	8.78	22.85
N-H	399.8	63.71	89.56	--
N-C	401.3	36.29	10.44.	--
